# Insights into the bioavailability of oceanic dissolved Fe from phytoplankton uptake kinetics

**DOI:** 10.1038/s41396-020-0597-3

**Published:** 2020-02-05

**Authors:** Yeala Shaked, Kristen N. Buck, Travis Mellett, Maria. T. Maldonado

**Affiliations:** 1 The Fredy and Nadine Herrmann Institute of Earth Sciences, Hebrew University of Jerusalem, Jerusalem, Israel; 2 Interuniversity Institute for Marine Sciences, Eilat, Israel; 3 College of Marine Science, University of South Florida, Tampa, FL USA; 4 Earth, Ocean and Atmospheric Sciences Department, University of British Columbia, Vancouver, BC Canada

**Keywords:** Biogeochemistry, Water microbiology

## Abstract

Phytoplankton growth in large parts of the world ocean is limited by low availability of dissolved iron (dFe), restricting oceanic uptake of atmospheric CO_2_. The bioavailability of dFe in seawater is however difficult to appraise since it is bound by a variety of poorly characterized organic ligands. Here, we propose a new approach for evaluating seawater dFe bioavailability based on its uptake rate constant by Fe-limited cultured phytoplankton. We utilized seven phytoplankton species of diverse classes, sizes, and provenances to probe for dFe bioavailability in 12 seawater samples from several ocean basins and depths. All tested phytoplankton acquired organically bound Fe in any given sample at similar rates (after normalizing to cellular surface area), confirming that multiple, Fe-limited phytoplankton species can be used to probe dFe bioavailability in seawater. These phytoplankton-based uptake rate constants allowed us to compare water types, and obtain a grand average estimate of seawater dFe bioavailability. Among water types, dFe bioavailability varied by approximately four-fold, and did not clearly correlate with Fe concentrations or any of the measured Fe speciation parameters. Compared with well-studied Fe complexes, seawater dFe is more available than model siderophore Fe, but less available than inorganic Fe. Exposure of seawater to sunlight, however, significantly enhanced dFe bioavailability. The rate constants established in this work, not only facilitate comparison between water types, but also allow calculation of Fe uptake rates by phytoplankton in the ocean based on measured dFe concentrations. The approach established and verified in this study, opens a new way for determining dFe bioavailability in samples across the ocean, and enables modeling of in situ Fe uptake rates by phytoplankton using dFe concentrations from GEOTRACES datasets.

## Introduction

Iron (Fe) exerts a strong influence on the biogeochemical cycles of carbon and other bioactive elements in the ocean, through control of phytoplankton growth [1–6]. To understand and model how Fe concentrations control phytoplankton physiology and ecology, we must evaluate the bioavailability of Fe to phytoplankton. Building on the solid foundation laid during 40 years of extensive research on Fe availability to laboratory cultures [7–11], we set out to evaluate the bioavailability of natural dissolved Fe (dFe) from different oceanic locations and depths by examining phytoplankton uptake kinetics.

Our approach of conducting uptake assays to determine the bioavailability of Fe bound to uncharacterized organic ligands in the surface ocean draws from the seminal studies of Hudson and Morel [12, 13] and Sunda and Huntsman [14, 15], who reported that several Fe-limited eukaryotic phytoplankton species acquire dissolved inorganic Fe (Fe′) at identical rates, at a given Fe concentration, when accounting for their cell-surface area (S.A.). The consistency in S.A. that normalized Fe uptake rate probably reflects the number of Fe transporters on the cell surface, which is limited by the size of the transporters and the available membrane area allocated for Fe acquisition [12]. The similarity in S.A.-normalized Fe uptake rate among eukaryotic phytoplankton species suggests that Fe in aquatic environments exerted significant selective pressure on phytoplankton to evolve Fe uptake mechanisms that operate at the optimal efficiency permitted by fundamental biophysical and chemical constraints [13, 14].

These findings were recently extended to additional eukaryotic and prokaryotic phytoplankton species by Lis et al. [16], who invoked the use of Fe uptake rate constants (k_in_ in units of L cell^−1^ d^−1^) rather than uptake rates to compare phytoplankton data from uptake assays with different Fe concentrations [Fe], where k_in _= uptake rate/[Fe]. Examining Fe uptake rate constants (k_in_ of Fe) of 25 different Fe-limited phytoplankton species, and accounting for their surface areas, they found a remarkable similarity among all species, implying that the bioavailability of Fe can be described with a single surface area-normalized uptake rate constant (k_in_/S.A. of Fe′) [16]. Similarly, uptake of Fe from strong organic Fe complexes—such as the siderophore Ferrioxamine B (FeDFB)—by various Fe-limited phytoplankton species was found to converge into a common surface area-normalized Fe uptake rate constant for each organic Fe complex tested (e.g., k_in_/S.A. of FeDFB). Interestingly, the k_in_/S.A. of different organic Fe complexes varied by 1000-fold, and the k_in_/S.A. of most complexes were much lower than those of inorganic Fe. These findings support previous data showing that inorganic Fe is more bioavailable than organically bound Fe, and that organic Fe complexes differ in their bioavailability to phytoplankton [9, 11, 17–19]. Therefore, the bioavailability of different Fe complexes—either organically bound (FeL) or inorganic Fe (Fe′)—can be defined and ranked according to their respective k_in_/S.A. values. These surface area-normalized uptake rate constants (k_in_/S.A.), obtained with phylogenetically diverse Fe-limited phytoplankton species, are robust tools for assessing bioavailability of Fe complexes since they remain unchanged in cells experiencing different degrees of Fe limitation [14, 16]. Yet, a significant drop in k_in_/S.A. was observed for Fe-sufficient cells [16] where different Fe transport mechanisms likely operate (low vs. high affinity) [11, 20, 21], and hence bioavailability of Fe complexes can be probed only with Fe-limited phytoplankton.

Here, we applied this approach to probe the bioavailability of Fe in seawater, where the vast majority of dFe is bound to a mixture of mostly uncharacterized organic ligands. Indeed, the speciation of dFe in seawater is typically determined by concentrations and stability constants of organic ligands, which are measured with electrochemical methods [22–24]. These measurements allow us to ascribe Fe-binding organic ligands (L_*i*_) to a given ligand class (*i* *=* 1, 2, 3, 4) based on their conditional stability constants ($K_{FeL_1,{{Fe}^\prime} }^{cond}$). The strongest ligands, those in the L_1_ ligand class, have the highest conditional stability constants (log $K_{FeL_1,{{Fe}^\prime} }^{cond}$ > 12), while L_2_ ligands have log $K_{FeL_2,{{Fe}^\prime} }^{cond}$ ranging between 11 and 12 [24]. Given that the nature of these dFe-binding organic ligands in seawater is largely unknown, we suggest treating the entire pool of dFe as the substrate for uptake, and describe the average bioavailability of this pool with a single surface area-normalized Fe uptake constant—k_in-app_/S.A. We added an apparent (i.e., “app”) term to the uptake constant to indicate that it probably reflects a mixture of Fe complexes, unlike the k_in_ that is drawn from a specific Fe complex [16].

Our study aimed at probing oceanic dFe bioavailability by using several cultured Fe-limited phytoplankton and seawater samples collected from a variety of oceanic regions and depths. dFe bioavailability in each seawater sample was assessed with a single kinetic term—k_in-app_/S.A.—established by multiple short-term Fe uptake experiments with different Fe-limited cultures. We carefully tested and verified this novel methodology, and examined the effects of photochemistry and Fe speciation on dFe bioavailability in these natural seawater samples.

## Methods

### Seawater sample collection

Seawater samples from different ocean basins (Table 1) were collected following GEOTRACES protocols [25] using trace-metal clean rosette systems outfitted with either GO-Flo (General Oceanics) or x-Niskin samplers (Ocean Test Equipment). Samples were filtered through 0.2 µm Acropak capsule filters and stored frozen (−20 °C) in trace-metal clean 500-mL fluorinated high-density polyethylene (FPE) bottles (Nalgene) until analyzed for Fe speciation at the University of South Florida. The FPE bottles for dFe speciation were cleaned by soaking first in a 1% soap (Citranox; Fisher) bath for 1 week, followed by at least a month in a 25% hydrochloric acid (HCl, trace-metal grade, Fisher); once clean, the bottles were rinsed and filled with Milli-Q (>18.2 MΩ·cm) until use [26]. Arctic seawater from Baffin Bay (BBA), Canadian Arctic Archipelago (CCA), and Beaufort Sea (BS) was collected as part of the Canadian GEOTRACES Arctic Program (legs 2 and 3, Aug–Sep 2015). North Pacific seawater (P16, P20 surface, and P20 deep) was collected in June 2017 from stations P16 and P20 during a Line-P cruise (GPpr07 GEOTRACES Process Study). Seawater from the Eastern Tropical South Pacific (EPZT surface, EPZT deep, and EPZT oxygen-depleted zone—ODZ) was collected as part of the US GEOTRACES program cruise GP16 (Eastern Pacific Zonal Transect) in late 2013. Seawater from the North Atlantic (GTNA, GEOTRACES North Atlantic) was collected as part of the US GEOTRACES program cruise GA03 (North Atlantic Zonal Transect) in 2011. Seawater from the eastern Gulf of Mexico (GOM) was collected aboard the R/V Weatherbird II using a surface towfish in June 2015. Seawater from the Southern Ocean (SO) was collected from the R/V/I/B Nathanial B. Palmer using a trace-metal clean rosette system in September 2016 (cruise NBP 1608).

**Table 1:** Description of the seawater samples analyzed in this study, including sampling location, depth of collection, iron concentration (dFe), and iron speciation results, including iron-binding organic ligand (L_1_ and L_2_) concentrations and log-conditional stability constants (log K).

Sample description		Ocean basin	Lat	Long	Depth	[dFe]^a^	[L_1_]	log K(L_1_)	[L_2_]	log K(L_2_)	[Fe′]	α′^b^	α^c^
Full	Abbreviated		°N	°E	m	(nM)	(nM)		(nM)		pM		
Baffin Bay	BBA	Arctic	71.4	−68.6	37	1.56	1.83	12.46	–	–	2.0	779	5278
Canadian Arctic Archipelago	CAA	Arctic	74.5	−80.6	44	0.97	1.79	12.06	–	–	1.0	941	2055
Beaufort Sea	BS	Arctic	75.8	−129.2	10	0.74	–	–	1.74	11.67	1.6	468	814
North Pacific P16	P16	Pacific	49.2	−134.4	25	0.58	–	–	1.83	11.69	0.9	612	896
North Pacific P20 Surface	P20_Surf	Pacific	49.3	−138.4	25	0.95	1.35	12.30	1.82	11.28	0.8	1145	3040
North Pacific P20 Depth	P20_Deep	Pacific	49.3	−138.4	800	0.97	–	–	3.23	10.91	5.3	184	263
Southern Ocean	SO	Southern Ocean	−61.1	−67.3	100	1.37	1.92	12.08	–	–	1.8	743	2308
Gulf of Mexico	GOM	Atlantic	26.0	−85.5	2	0.60	–	–	1.81	11.79	0.8	746	1116
Atlantic Zonal Transect	GTNA	Atlantic	26.1	−44.8	100	0.63	0.79	12.39	0.93	11.26	1.1	591	2108
Equatorial Pacific Oxygen Depleted Zone	EPZT_ODZ	Pacific	−12.0	−77.7	20	1.67	1.48	12.57	2.73	11.08	5.5	302	5827
Equatorial Pacific Surface	EPZT_Surf	Pacific	−12.0	−94.0	2, 20	0.46	0.78	12.35	1.22	11.39	0.5	955	2046
Equatorial Pacific Depth	EPZT_Deep	Pacific	−12.0	−89.0	3000	1.17	–	–	3.01	11.39	2.3	513	739

Ligand classes (L_1_, L_2_) were defined by measured conditional stability constants; the notation of “–” was used for ligand classes not detected in the samples. Dissolved inorganic Fe (Fe′) and complexation capacity of free ligands (α′) and total ligands (α) were calculated from the speciation data.

^a^Ambient dissolved Fe (dFe) plus 0.4 nM ^55^Fe.

^b^$$\alpha ^\prime = {\sum} {\left[ {eL_i} \right]} \times K_{FeL_i,{{Fe}^\prime} }^{cond}$$

^c^$$\alpha = {\sum} {[L_i] \times K_{FeL_i,{{Fe}^\prime} }^{cond}}$$

### Phytoplankton choice, culturing, and growth monitoring

Seven phytoplankton species from four classes were cultured in this study (Table 2). The species chosen represent dominant phytoplankton phyla in coastal- and open-ocean waters, and encompass a large size range. Cultures were obtained from the northeast Pacific Culture Collection (NEPCC, University of British Columbia, Vancouver, Canada) and the Center for Culture of Marine Phytoplankton (CCMP, Bigelow Laboratory, West Boothbay Harbor, ME, USA). Sterile, trace-metal‐clean techniques were used throughout the experiments. All culture bottles and plasticware used to prepare the media were soaked in a dilute Extran detergent for 48 h, rinsed with deionized water, soaked in 10% nitric acid for 48 h, rinsed six times with ultrapure water (18 MΩ·cm; Milli-Q, Millipore), and finally microwave-sterilized before use. Cultures were grown in 28-mL polycarbonate tubes at 19 ± 1 °C under a continuous saturating light level of 175 μmol quanta m^−2^ s^−1^ as described in Maldonado et al. [20]. Briefly, growth media was based on station Papa seawater collected during a Line-P cruise and filtered (0.2 μm, AcroPack) onboard according to GEOTRACES protocols. Station Papa seawater was enriched with filter-sterilized (0.2 µm, Acrodisc) nitrate (300 μmol L^−1^ NO_3_^−^), phosphate (10 μmol L^−1^ PO_4_^3–^), and silicic acid (100 μmol L^−1^ H_4_SiO_4_) that were first passed through a chromatography column (Bio-Rad) containing Chelex® ion-exchange resin (Bio-Rad) to remove any trace metals (“chelexed”). Prior to use, this basal medium was microwave-sterilized and amended with vitamins (biotin, thiamine, and B_12_), and a mix of trace metals (without Fe) buffered with 100 μmol L^−1^ EDTA (ethylene-diamine-tetraacetic acid). Finally high Fe (1.37 μmol L^−1^; pFe19) and low Fe (12.5 nmol L^−1^; pFe21) media were generated by adding Fe complexed by EDTA (1:1.5 Fe:EDTA ratio). Cultures were constantly maintained in the exponential phase by serial dilutions into fresh medium as needed, and growth rates were monitored daily by measuring in vivo chlorophyll *a* fluorescence (Turner 10-AU Fluorometer). Cultures were first grown in high Fe and then gradually acclimated to low Fe. Specific growth rates (*µ*) were determined as per day (d^−1^) from linear regressions of the natural log of in vivo fluorescence versus time during the exponential growth phase of acclimated cells, after confirming a proportional relationship between fluorescence and cell concentration. Cultures were considered acclimated when growth rates in successive transfers varied by less than 15% (typically 4–5 transfers). Cell concentrations and diameters were measured using a Coulter Z2 Particle Count and Size Analyzer (Beckman Coulter Inc.). Cell-surface area and volume were calculated, assuming that the cells were spherical, for all cultures except *Prorocentrum micans* (PM), where a prolate sphere equation was applied. Coulter counter-based surface area estimates were refined and confirmed by light and epi-fluorescence microscopic observations.

**Table 2:** Description of the phytoplankton species cultured in this study under iron-limited conditions in order to probe the bioavailability of dissolved iron (dFe) in natural seawater.

Phylum	Species			Environment	Fe-limited cell size		Fe-limited growth rate	
	Full name	Abbreviated	Strain		Diameter (µm)	S.A. (µm^2^)	µ (d^−1^)	% from µ_max_
Dinoflagellata	*Prorocentrum micans*	PM	CCMP 692	Coastal	22–25(H) 11–13(D)	1000 1300	0.24–0.28	66–78%
Bacillariophyta	*Thalassiosira weissflogii*	TW	CCMP 1047	Coastal	10.8–11.2	364–392	0.63–0.69	75–82%
Bacillariophyta	*Thalassiosira oceanica*	TO	CCMP 1003	Open ocean	4.9–5.2	75–84	0.83–0.85	67–70%
Bacillariophyta	*Thalassiosira pseudonana*	TP	NEPCC 58	Open ocean	4.7–5.2	70–78	0.73–0.75	65–67%
Haptophyta	*Chrysochromulina polylepis*	CP	NEPCC 242	Coastal/ open	3.9–4.1	47–53	0.33–0.39	41–49%
Haptophyta	*Phaeocystis pouchetii*	PP	NEPCC 225	Open ocean	3.9–4.3	48–58	0.50–0.55	59–65%
Chlorophyta	*Micromonas sp*.	Mic	CCMP 2709	Open ocean	1.4–2.3	6–16	0.33–0.39	50–59%

“Environment” refers to the oceanic ecosystem where these species typically grow. Cells diameter was measured with a Coulter Counter and was converted to surface area (S.A.) assuming a spherical shape (except for *Prorocentrum micans*, where a prolate sphere equation was applied). A range of growth rates is reported for the cultures that were kept in exponential growth under Fe-limiting conditions for at least 25 generations prior to the uptake experiments.

### Short-term Fe uptake experiments

The bioavailability of dFe in the different seawater samples was examined by an extensive series of short-term uptake assays with Fe-limited cultures using low, sub-nanomolar concentrations of the radiotracer ^55^Fe. The radioisotope was purchased from Perkin Elmer as an acidic ^55^FeCl_3_ solution with a specific activity 47 mCi mg Fe^−1^. Additions of radioisotope into the assay consider the total amount of Fe in the stock (including non-radiolabeled Fe), but for simplicity are noted as ^55^Fe only. Contamination with non-radiolabeled Fe was minimized by following stringent trace-metal clean protocols and conducting all assays within a laminar flow hood. Concentrations of free organic ligands in all samples (Table 1) were higher than the added 0.4 nM ^55^Fe, enabling the radiotracer to be complexed by the ambient ligands. To allow enough time for equilibration between the radiotracer and the ambient organic ligands, samples were thawed and spiked with ^55^Fe, 24 h prior to the uptake assays. ^55^Fe loss to the bottle walls was prevented by pre-mixing the ^55^Fe stock with a low EDTA concentration (1:1.2 Fe:EDTA ratio). The exchange of ^55^Fe between the added ^55^FeEDTA and the natural ligands in seawater was examined by Mellet et al. 2018, who found that a period of 24 h was sufficient for equilibration between FeEDTA and natural ligands [27]. Briefly, 0.2 nM FeEDTA (1:1.2 Fe:EDTA ratio) was added to NE Pacific seawater sample from a depth of 20 m, and the speciation of Fe was characterized prior to and 24 h after FeEDTA addition by voltammetry (see “Methods”). The addition of FeEDTA resulted in a drop of ~0.4 nM in the measured free ligand concentrations [L], suggesting that Fe dissociated from the FeEDTA complex and was complexed by the free natural ligands (Supplementary Fig. S2). For the short-term Fe uptake assays, each ^55^Fe spiked water type was split to 80–150 mL aliquots and placed in clean polycarbonate bottles. Mid-exponential Fe-limited cultures were gently filtered on acid-cleaned polycarbonate membrane filters of appropriate pore size and carefully washed with chelexed seawater to remove left-over growth media, while keeping the membranes wet at all times. Cells were then resuspended in the ^55^Fe spiked aliquots and incubated at room temperature (20–22 °C) under dim laboratory light for 4 to 6 h. Every 1–2 h duplicate subsamples were withdrawn and filtered onto polycarbonate membranes and rinsed with 0.2-µm filtered-Ti(III)EDTA-citrate reagent to remove extracellularly absorbed Fe [28]. The ^55^Fe activity accumulated by the cells and in the tested solution was measured in a scintillation counter (Beckman-Coulter LS 6500; Beckman Counter Inc.) using Ecolite + scintillation cocktail (Fisher). Cell densities in the experiments were similar or slightly more concentrated than those in the original exponential cultures, ranging between ~3–5 × 10^3^ cells mL^−1^ in the largest species PM and 1–2 × 10^6^ cells mL^−1^ in the smallest species *Micromonas sp*. (Supplementary Table S2). Cell concentrations and diameters in each bottle were measured during the assay using a Coulter Counter (Coulter Z2 Particle Count and Size Analyzer). Fe uptake rates (mol Fe cell^−1^ d^−1^) were calculated from the linear regression of the accumulated cellular Fe as a function of incubation time, considering the ambient dFe concentrations. Fe uptake by killed cells was negligible and so was the ^55^Fe loss to the bottle walls (total activity remained unchanged throughout the experiment). Due to limited sample volume, not all experiments were run in duplicates. A few uptake assays were conducted outdoors to examine the effect of sunlight on dFe availability, using UV transparent acid-cleaned 1‐L Cubitainers (Fisher). To minimize the exposure time of the cells to the strong natural sunlight (~1000 μmol quanta m^−2^ s^−1^), the seawater (without cells) was first exposed to sunlight for 1–2 h. Washed cells were then quickly suspended in these pre-exposed samples and placed outdoors (but avoiding direct sunlight) and sub-sampled every 30–45 min for up to 1–2 h. Parallel outdoors dark controls were performed and compared with the sun-exposed rates, to account for container and temperature effects.

### Total dissolved Fe and Fe speciation

Dissolved Fe and Fe speciation data from the GEOTRACES samples (EPZT and GTNA) were previously published along with the methods used [22, 29]. For the remaining samples, an aliquot of seawater was poured into an acid clean 125 mL FPE bottle and acidified to pH 1.8 (0.024 M Optima grade HCl, Fisher). Dissolved Fe in the acidified sample was preconcentrated on a Nobias 1 A resin using the commercially available seaFAST (ESI) system, which facilitates preconcentration of trace metals from seawater samples into 1 N quartz-distilled nitric acid eluent. The eluent was then measured on a Thermo Scientific Element XR inductively coupled plasma mass spectrometer via method of standard addition; standard curves constructed in low metal and GEOTRACES reference samples were also processed on the seaFAST system to verify results. Fe speciation in the remaining unacidified seawater was determined using competitive ligand exchange-adsorptive cathodic stripping voltammetry (CLE-AdCSV; [29]). Briefly, fifteen 10 mL aliquots of the samples were buffered to pH 8.5 with a borate-ammonium buffer and titrated with thirteen additions of dFe (0.1–10 nM, with two blanks). Following overnight equilibration of the Fe additions, the same aliquots were spiked with 25 µM salicylaldoxime as a competing ligand, allowed to equilibrate again for at least 1 h, and the resulting concentration of Fe bound to salicylaldoxime was measured by AdCSV on a BAS*i* hanging mercury drop electrode. The titration data generated from this approach was then interpreted using the measured dFe concentrations in the freely available software program ProMCC [30] to obtain Fe-binding ligand concentrations, their stability constants and the 95% confidence interval of each result. Titrations were modeled for one- and two-ligand classes with the selection of a model based on the same criteria used for GEOTRACES data sets, following best practices from intercomparisons of complexometric titration data [31], and using the visualization of results provided in ProMCC to determine the best fit to the titration data. Information on speciation data, calculations and errors appear in Supplementary Table S1.

### Data analysis and statistics

Iron uptake rates were converted to per cell uptake rates constants (k_in-app_) based on the measured dFe in the sample (ambient + added ^55^Fe) and cell numbers, and were normalized to the cell-surface area, yielding the measure of dFe availability—k_in-app/_S.A. One-way repeated-measures analysis of variance was performed to test whether the average k_in-App/_S.A. was indeed significantly different among seawater samples. Pearson correlations between the average k_in-app_/S.A. values for each seawater sample and the Fe speciation parameters we determined were performed to explore the link between Fe speciation and dFe bioavailability.

## Results

We evaluated dFe bioavailability in 12 water samples from different locations and depths (Table 1; Supplementary Table S1) by conducting ~85 short-term Fe uptake experiments with Fe-limited cultures of 7 different phytoplankton strains varying in size, taxa, and isolation site (Table 2). From these measured Fe uptake rates, we calculated the dFe uptake rate constant for each experiment, for a specific phytoplankton species and seawater (SW) sample:

1 $$\,\,{\mathrm{k}}_{{\mathrm{in - app}}}\left( {{\mathrm{L}}\;{\mathrm{cell}}^{ - {\mathrm{1}}}{\mathrm{d}}^{ - {\mathrm{1}}}} \right) 	=\, {\mathrm{uptake}}\;{\mathrm{rate}}\left( {{\mathrm{mol}}\;{\mathrm{Fe}}\;{\mathrm{cell}}^{ - 1}{\mathrm{d}}^{ - 1}} \right)\\ 	{\hskip 10pt}/\,{\mathrm{SW}}\;{\mathrm{dFe}}\;{\mathrm{concentration}}\left( {{\mathrm{mol}}\;{\mathrm{Fe}}\;{\mathrm{L}}^{ - 1}} \right)$$

These phytoplankton- and seawater-specific k_in-app_ values were then normalized to their respective cell S.A. to derive our dFe bioavailability proxy:

2 $${\mathrm{Bioavailability}}\;{\mathrm{of}}\;{\mathrm{SW}}\;{\mathrm{dFe}}\left( {{\mathrm{L}}\; {\upmu} {\mathrm{m}}^{ - 2}{\mathrm{d}}^{ - 1}} \right) 	= {\mathrm{k}}_{{\mathrm{in - app}}}\left( {{\mathrm{L}}\;{\mathrm{cell}}^{ - 1}{\mathrm{d}}^{ - 1}} \right)\\ \quad 	{\hskip 10pt}/\,{\mathrm{S}}{\mathrm{.A}}{\mathrm{.}}\left( {{\upmu}{\mathrm{m}}^2{\mathrm{cell}}^{ - 1}} \right)$$

### Establishing surface area-normalized Fe uptake rate constant—k_in-app_/S.A.—as a proxy of dFe bioavailability in natural seawater samples

**Fig. 1:**
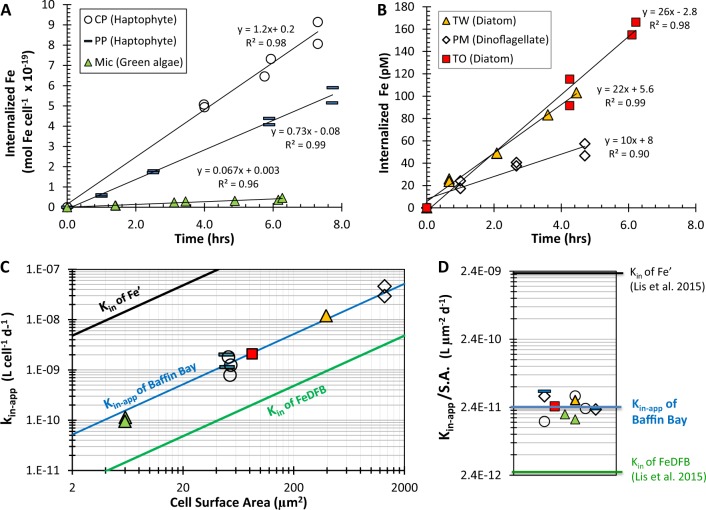
Evaluation of the bioavailability of dissolved iron (dFe) in water from Baffin Bay in the Arctic. **a**, **b** Dissolved Fe uptake by Fe-limited phytoplankton species measured under stringent trace-metal clean protocols and internalized Fe plotted per cell (**a**) or as total internalized Fe (**b**). **c**, **d** Calculated Fe uptake rate constants for Baffin Bay water—k_in-app_—the measure of dFe bioavailability as probed by Fe-limited phytoplankton species. Plotting k_in-app_ versus cellular surface area (S.A.) in a log-log plot yielded a single line (**c**), implying that all phytoplankton probe dFe bioavailability similarly when normalized to surface area (S.A.). Hence, a single kinetic term, k_in-app_/S.A., can describe the dFe bioavailability in these waters (**d**). The bioavailability of dFe in Baffin Bay is intermediate between that of inorganic Fe (Fe′) and that of Fe bound to the strong siderophore desferrioxamine B (FeDFB), determined previously. Each phytoplankton strain is labeled in a unique symbol and color that is consistent across all panels.

Having proposed a novel methodology for probing dFe bioavailability in natural waters, we first present raw data from a single seawater sample (Baffin Bay) to discuss our experimental design and interpretation of results. The availability of dFe in Baffin Bay water (BBA, Table 1) was tested using six different Fe-limited phytoplankton species (Table 2) in 11 individual uptake experiments, using ^55^Fe and stringent trace-metal clean protocols (Fig. 1; Supplementary Table S2). In each experiment, the internalization of ^55^dFe by the cells was monitored over 3–6 h by repeated filtrations of subsamples (Fig. 1a, b; Supplementary Table S2). These cellular ^55^Fe uptake rates (e.g., Fig. 1a) were divided by the total dFe concentration in the assays ([dFe] + 0.4 nM ^55^Fe), to derive the Fe uptake rate constants—k_in-app_—for each phytoplankton species (Eq. 1).

In order to calculate k_in-app_ by dividing Fe uptake rate by dFe concentration—mostly composed of organically bound Fe—and not by the inorganic Fe concentration (Fe′), it is necessary to demonstrate that the cells are indeed accessing Fe from the organic Fe pool. To do this, we first estimated dissociation rate constants of the organically bound Fe complexes (*k*′_*d*_*)* in our natural seawater samples containing L_1_ and/or L_2_ organic ligands (Supplementary Table S3), using the formation rate constants (*k*′_*f*_) determined in natural seawater by Croot and Heller [32] and our measured log $K_{FeL_i,{{Fe}^\prime} }^{cond}$ (Supplementary Table S3). For example, for Baffin Bay we calculated a dissociation rate constant—*k*′_*d*_—of 1.1–2.4 × 10^–7^ s^−1^ [calculated from $K_{FeL_i,{{Fe}^\prime} }^{cond}$ = *k*′_*f*_/*k*′_*d*_, where log $K_{FeL_1,{{Fe}^\prime} }^{cond}$ = 12.46 and *k*′_*f*_ *=* 3.2–6.9 × 10^5^ M^−1^ s^−1^]. Then, using these dissociation rate constants and the measured [dFe], we estimated the maximum supply of Fe′ to the cell surface for biological uptake; which in the case of Baffin Bay it was 1.3 pmol Fe L^−1^ h^−1^ (Supplementary Table S3). This supply rate of Fe′ due to dissociation of Fe from organic Fe complexes (FeL) is significantly slower than the Fe uptake rates measured under very dim light (ranged from 7 to 32 pmol Fe L^−1^ h^−1^; Fig. 1b, Supplementary Table S2), implying that the cells were indeed accessing Fe from the organic Fe pool. Moreover, the Fe′ concentration initially present in Baffin Bay seawater (2 pmol L^−1^, Table 1) makes a negligible contribution to the 48–270 pmol L^−1^ Fe internalized by the cells throughout the experiments (Fig. 1b; Supplementary Table S2). Similar findings were determined for all water types, except for one (Supplementary Tables S2–S4). Hence, we deduced that phytoplankton were indeed accessing Fe from organic complexes in the dissolved Fe pool, and thus the k_in-app_ should be calculated by dividing Fe internalization rates by concentration of dFe, most of which (>99%) is organically bound [24]. Note that our kinetic-based account of Fe uptake from Fe– organic ligand complexes does not specify or confine the transport mechanism at play. The reported mechanisms include reductive release of Fe(II) from the Fe–organic ligand complex [11, 17–19, 33], ternary complex formation and possible transfer of Fe from the organic to cell-surface ligand [34, 35], and internalization of the whole complex [36, 37], any of which may act simultaneously or separately.

Another important criterion for calculating k_in-app_ is that the rate of Fe uptake has to remain constant for the duration of the experiments (i.e., linear regression between internalized Fe and time). Indeed, the vast majority of the Fe uptake rates were linear (R^2^ > 0.85) and had an intercept near the origin (e.g., Fig. 1a, b). In a few experiments (<10%), we observed slower Fe internalization in the last time point, which was then excluded from the calculated rates of Fe uptake. For uptake to remain linear over the course of the experiment it is important to ensure that the overall Fe concentration acquired by the cells does not approach the total available Fe pool; this was accomplished using low cell densities in the experiments (Supplementary Tables S2, S4). The internalized Fe at the end of our uptake assays with the different water samples ranged from 2 to 270 pM (Supplementary Tables S2, S4), and at most, accounted for 22% of the total dissolved Fe concentration.

Next, we examined if all tested phytoplankton  species equally probe the bioavailability of dFe in a single water sample by plotting k_in-app_ against cell S.A., or by normalizing k_in-app_ to S.A. For Baffin Bay water, all 11 k_in-app_ values fall on a single line when plotted against S.A. (Fig. 1c) and can thus be normalized to their respective cellular S.A. to yield an average k_in-app_/S.A. of 2.6 × 10^−11^ ± 8.5 × 10^−12^ L µm^−2^ d^−1^ (Fig. 1d; Supplementary Table S2). This surface area-normalized k_in-app_ describes the average bioavailability of the dissolved Fe in Baffin Bay seawater, and it fits within the “bioavailability envelope” of Lis et al. [16] (black and green lines in Fig. 1c, d).

### Evaluating dFe bioavailability in different seawater samples

**Fig. 2:**
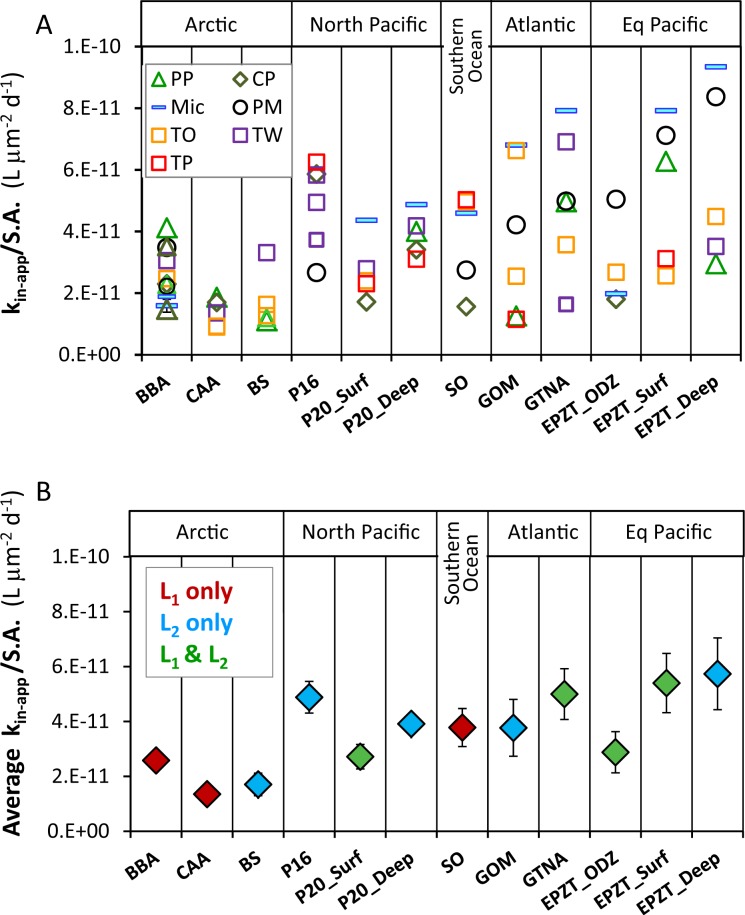
Bioavailability of dissolved iron (dFe) in the 12 seawater types probed with seven iron-limited phytoplankton species under dim laboratory light. **a** Individual dFe bioavailability proxies (k_in-app_/S.A.) in all tested water types (abbreviated on the *x* axis), derived from Fe uptake rates of various Fe-limited phytoplankton (abbreviated in the legend). **b** Average dFe bioavailability (average k_in-app_/S.A.) of all phytoplankton species as a function of water types, colored according to the classes of ligands present in each sample. Error bars are 1 standard deviation of the average. Phytoplankton species: *Prorocentrum micans* (PM), *Thalassiosira weissflogii* (TW), *Thalassiosira oceanica* (TO), *Thalassiosira pseudonana* (TP), *Chrysochromulina polylepis* (CP), *Phaeocystis pouchetii* (PP), and *Micromonas sp*. (MIC). Seawater samples: Arctic—Baffin Bay (BBA), Canadian Arctic Archipelago (CCA), Beaufort Sea (BS); North Pacific—Line-P stations—P16, P20 surface (P20_Surf), P20 depth (P20_Deep); Southern Ocean (SO); Atlantic—Gulf of Mexico (GOM), GEOTRACES GA03 Atlantic Zonal Transect (GTNA); Equatorial Pacific—GEOTRACES GP16 Oxygen Depleted Zone (EPZT_ODZ), surface (EPZT_Surf), and depth (EPZT_Deep).

To evaluate dFe bioavailability in a variety of seawater samples, in addition to Baffin Bay, we present the dissolved Fe bioavailability proxy—k_in-app_/S.A.—for all 12 water types in two formats: the value for individual experiments for each seawater sample and phytoplankton (Fig. 2a); and the average dFe bioavailability value for each water type (Fig. 2b). We also present all k_in-app_/S.A. values in a supplementary table organized according to the phytoplankton species with which they were probed (Supplementary Table S5). In the individual experiments (Fig. 2a), it is evident that in some water types, all k_in-app_/S.A. values obtained by different phytoplankton species agree well with each other (8–14% standard error), while in other samples k_in-app_/S.A. values are more scattered (18–27% standard error). In some water types, *Micromonas Sp*. (Mic) and *Prorocentrum micans* (PM), yield the highest k_in-app_/S.A., but this trend is reversed in other samples where *Thalassiosira weissflogii* (TW), *Thalassiosira pseudonana* (TP) and *Phaeocystis pouchetii* (PP) yield maximum values (Fig. 2a). Comparison of k_in-app_/S.A. values from all water types across phytoplankton species (Supplementary Table S5), did not show any statistical differences among them. The lack of systematic differences in the dFe bioavailability parameter among phytoplankton species for a single seawater sample (Fig. 2a; Supplementary Table S5) suggests that the surface area-normalized Fe uptake constants of eukaryotic phytoplankton are similar, in line with previous studies [11, 16, 38]. This lack of systematic differences in k_in-app_/S.A. among tested phytoplankton strains does not necessarily imply that they all use similar uptake mechanisms, rather it indicates that their uptake mechanisms operate at the optimum efficiency permitted by fundamental physical, chemical or biochemical factors [13, 14]. These findings suggest that multiple or any Fe-limited eukaryotic phytoplankton species can be used to probe dFe bioavailability in a water sample, and that the grand average Fe bioavailability value derived from a series of experiments with a single or several different phytoplankton species can be used to estimate the dFe bioavailability in that water sample. In future experiments employing this methodology, we recommend the use of phytoplankton with rigid cell walls and simple shape, such as centric diatoms, to ease experimental manipulations and calculations.

Moving from individual experiments (Fig. 2a) to average values (Fig. 2b), we performed one-way repeated-measures analysis of variance to test whether the average bioavailability of dFe was indeed significantly different among seawater samples. This analysis revealed that dFe bioavailability proxy (k_in-app_/S.A.) was significantly different among seawater samples (*p* < 0.01). The bioavailability of dFe among water types differs by ~four-fold, from the least bioavailable dFe in the Canadian Arctic Archipelago (CAA) to the most bioavailable dFe in the Equatorial Pacific Deep (EPZT_Deep; Fig. 2b). All three Arctic samples had relatively low dFe bioavailability, whereas some of the Equatorial and North Pacific samples had relatively high dFe availability. While seawater temperature and irradiance will affect dFe bioavailability in surface oceanic waters, in our laboratory experiments these variables were constant. Hence, the differences in dFe bioavailability reported here likely reflect differences in the chemical speciation of Fe in seawater. To explore the link between Fe speciation and dFe bioavailability, we performed Pearson correlations between the average k_in-app_/S.A. values for each seawater sample and the Fe speciation parameters we determined, and are present in Table 1 and Supplementary Table S1. These include: Fe concentrations ([dFe] and [Fe′]), ligands concentrations ([L_1_], [L_2_], [L_total_] (i.e. sum of both ligands), and [eL] (i.e. excess ligands not bound by Fe)) and complexation capacity of total ligands (α) and free ligands (α′). The statistical analysis did not reveal any significant correlations (*p* > 0.05). In addition, visual examination of samples with different ligand types (L_1_ or L_2_ only, or both), did not reveal consistent trends between k_in-app_/S.A. and [dFe], calculated FeL dissociation rate, complexation capacity of the free Fe-binding ligands in seawater (α′), or excess ligands [eL] (Supplementary Fig. S1).

### Effect of photochemistry on dFe bioavailability

**Fig. 3:**
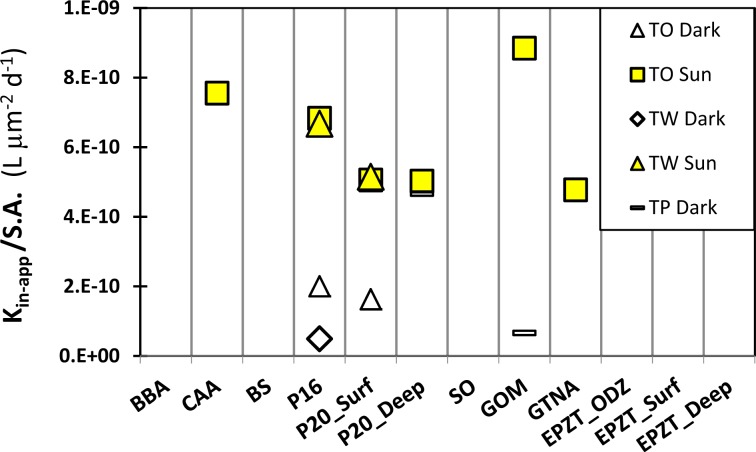
Effect of photochemistry on bioavailability of dissolved iron (dFe) in selected seawater types as probed with iron-limited diatoms during outdoor incubations. Diatoms species: TW, *Thalassiosira weissflogii*; TO *Thalassiosira oceanica*; TP, *Thalassiosira pseudonana*. Illuminated samples are noted as Sun, while covered samples are noted as Dark. See Table 1 for seawater type descriptions.

All laboratory experiments were conducted under room-dim lights, not inducing photochemical production of Fe′ from organically bound Fe. In the upper ocean, light and temperature influence the speciation of dFe and are thus likely to affect dFe bioavailability [36]. To examine the effect of photochemistry on our measured dFe bioavailability, we conducted a limited set of outdoor uptake experiments with three species of diatoms and six water types. In all the experiments, exposure to sunlight significantly increased the dFe bioavailability, with an average increase of six-fold in k_in-app_/S.A. relative to the outdoor, dark controls (Fig. 3, sunlight/dark ratio ranging from 1 to 13).

In order to compare our experiments with published in situ experiments, we converted the Fe uptake rates reported in these studies to k_in-app_/S.A. (Supplementary Table S6). In the Southern Ocean [18] and the California upwelling system [27], sunlight resulted in a 3- to 27-fold increase in dFe availability to natural phytoplankton, similar to the light enhancement in our experiments (1–13-fold, Fig. 3). In addition to documenting a positive effect of photochemistry on natural dFe availability, our outdoor experiments yielded k_in-app_/S.A. values that agree well with other sunlight-exposed experiments [33, 37] (Supplementary Table S6), suggesting that our dFe bioavailability estimates can be generalized and implemented in models. Our findings are in agreement with previous studies documenting that in situ Fe ligands are photolabile, implying that photochemistry in surface waters may play a significant role in Fe acquisition from the dissolved organic Fe pool by oceanic planktons [18, 27].

## Discussion

**Fig. 4:**
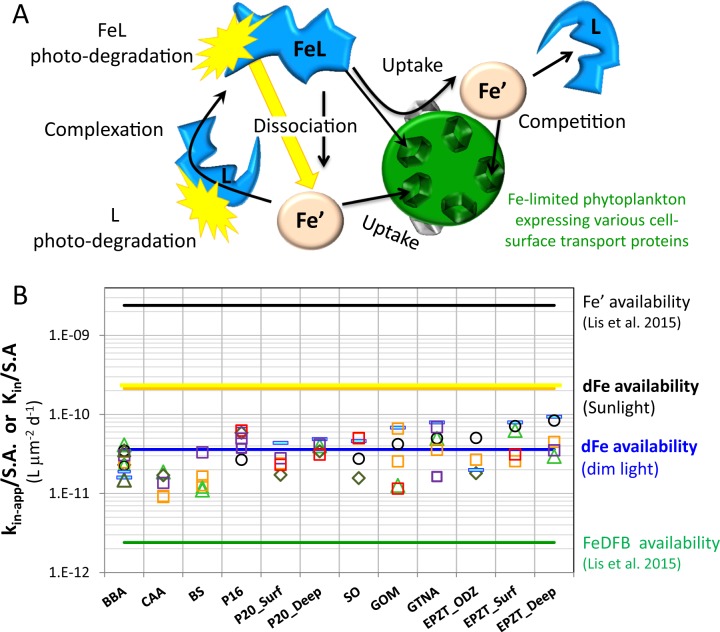
Summary and synthesis. **a** Schematic representation of the major reactions at play, which govern the rate of uptake of iron (Fe) from inorganic (Fe′) and organic (FeL) complexes by Fe-limited phytoplankton, and subsequently determine the dFe bioavailability proxy—k_in-app_/S.A. **b** Summary of all data evaluating dFe bioavailability across all water types from this study in dim laboratory light and in outdoor full light, plotted within the framework of the bioavailability envelope of [16]. Symbols represent individual phytoplankton strains as in Fig. 2a. See Table 1 for seawater type descriptions.

To assist synthesis, we provide a schematic outline of the major reactions at play that govern the rate of uptake by Fe-limited phytoplankton, and subsequently determine the dFe bioavailability proxy—k_in-app_/S.A. (Fig. 4a). These include acquisition of Fe’ and FeL via different pathways, dark dissociation of organically complexed Fe (FeL), and competition for Fe′ between free organic ligands (L) in seawater and cell-surface transporters/proteins. Photochemistry strongly enhances FeL dissociation, generating highly available Fe′, and photodegrading Fe-binding ligands, possibly decreasing Fe′ complexation and competition with the cell transporters (Fig. 4a). We will further explore these processes in an oceanographic context in the next paragraphs.

First, we discuss our experimental data within the context of the “bioavailability envelope” proposed by Lis et al. [16]. So far, we have highlighted a ~four-fold difference in dFe bioavailability among water types (Fig. 2). Nonetheless, considering that Fe bioavailability changes by 3 orders of magnitude between inorganic Fe (Fe′) and Fe bound to very strong siderophores like FeDFB (green and black lines in Fig. 4b), the bioavailability of dFe in the different water samples is, in fact, surprisingly similar. We can thus average the data from all of our experiments (blue line in Fig. 4b), and compare this grand average dFe bioavailability of oceanic seawater with that of specific Fe-binding ligands. The average k_in-app_/S.A. of all indoor experiments is 3.6 ± 0.25 × 10^−11^ L µm^−2^ d^−1^, and it falls within the lower range of the “bioavailability envelope”, closer to the bioavailability of Fe bound to model siderophores such as FeDFB rather than to Fe′.

This average dFe bioavailability (k_in-app_/S.A.) is 15-fold higher than that of FeDFB (2.4 × 10^−12^ L µm^−2^ d^−1^). In samples containing only L_1_ ligands, the elevated dFe bioavailability is consistent with their lower conditional stability constants (log $K_{FeL_i,{{Fe}^\prime} }^{cond}$ = 12.1–12.5, Table 1) compared with DFB (log $K_{FeDFB,{{Fe}^\prime} }^{cond}$ > 13; ref. [39]), and likely indicates that phytoplankton can access Fe bound to other strong organic ligands in the L_1_ class. This is reasonable because hydrophilic ferrioxamine siderophores in the ocean are found in only pM concentrations [40, 41] relative to the nM concentrations of strong Fe binding ligands (L_1_) detected with electrochemical methods. In samples containing both ligand classes (L_1_ and L_2_), ligand exchange between the weaker L_2_ ligands and Fe transporters at the cell surface may facilitate uptake. Indeed, phaeophytin, protoporphyrin IX, and humic acids (log $K_{FeL_i,{{Fe}^\prime} }^{cond}$ = 11, 11.9, and 11.6, respectively) [42, 43] have log $K_{FeL_i,{{Fe}^\prime} }^{cond}$ values similar to those in our L_2_ ligand class, and previous studies have shown that some of these Fe chelates are more readily available to phytoplankton than FeDFB [18]. Given the elevated oceanic dFe availability compared to FeDFB, we highly recommend incorporating additional strong Fe-binding ligands in uptake experiments.

Our average dFe bioavailability (k_in-app_/S.A.) is 70-fold lower than Fe′ (2.4 × 10^–9^ L µm^−2^ d^−1^), but when the samples were exposed to sunlight, dFe bioavailability increased by ~6-fold (Figs. 3 and 4b). Given the higher availability (by 1000-fold) of Fe′ relative to Fe bound to strong organic ligands, like siderophores [16], we suspect that this enhancement in dFe bioavailability is due to rapid formation of Fe′ in the presence of sunlight, as demonstrated for naturally occurring marine siderophores [44]. The processes at play are probably photoreduction of organically bound Fe(III) and formation of highly bioavailable inorganic Fe [44, 45]. Such photoenhancement of dFe bioavailability may be very pronounced at low temperatures [46], which retard the oxidation of Fe(II) to the more insoluble Fe(III) species [47], thus increasing the steady-state concentration of the dissolved Fe′ pool. Photochemistry was also shown to degrade the L_1_ class ligands [23], and thus may enhance bioavailability by decreasing the competition for inorganic Fe between free ligand in seawater and ligands-like components involved in phytoplankton Fe uptake mechanisms. A positive effect of light on phytoplankton physiology is less likely, because Fe uptake is not light dependent, and our experiments were short (1–3 h) relative to the time required to synthesize de-novo proteins by eukaryotic phytoplankton. The data presented here, showing an increase in dFe availability when samples are exposed to sunlight (Figs. 3 and 4), suggest that photochemistry in surface waters plays a significant role in Fe acquisition from the dissolved organic Fe pool by oceanic phytoplankton, as previously documented [18, 27].

Another factor that can decrease  the bioavailability of dFe is the presence of excess ligands that can compete with the cells for free inorganic Fe [Fe(II) or Fe(III)] generated biologically during the uptake processes [9, 11] or by photochemical reactions [18, 27]. Such competition occurs if the complexation capacity of the free Fe-binding ligands in seawater (α′) is higher than that of cell-surface ligand-like components of Fe acquisition mechanisms (herein Y). To illustrate this point, we calculated for our experiments the complexation capacity of the free cell-surface associated ligands in Fe transport, α_Y_′ (where α_Y_′ = [Y] x$K_{FeY,{{Fe}^\prime} }^{cond}$) and compared it with α′. For this calculation, we assumed that Fe uptake and speciation are in pseudo-equilibrium (given the low cell densities, low light conditions, and the presence of excess, strong organic Fe-binding ligands), and used published estimates for the diatom *TW* [12] (see Supplementary Table S7 for details). Our calculated complexation capacity of the free cell-surface associated ligands involved in Fe transport during our experiments (α_Y_′, ranged 0.1–1.0) is orders of magnitude lower than that of the Fe-binding ligands in seawater (α′, ranged 184–1145, Table 1). This implies that in seawater where cell densities are similar and even lower than those in our experiments, free organic ligands may compete effectively for inorganic Fe with ligand-like enzymes or Fe transporters at phytoplankton cell surfaces.

The approach tested and verified in this paper is useful for (a) experimentally probing dFe availability in natural seawater by using cultured, Fe-limited phytoplankton, (b) predicting dFe bioavailability in oceanic regions, and c) modeling in situ phytoplankton Fe uptake rates in the ocean. Acknowledging the high complexity of the studied system, comprising multiple organic ligands binding Fe in seawater (in the L_1_ and L_2_ classes), as well as multiple Fe acquisition mechanisms in phytoplankton [11, 20, 48, 49], we postulate that our approach is an important practical solution that can advance our predictive capability of the role of iron in determining phytoplankton growth in the ocean.

For modelers, our rate-constant based approach provides a very simple and direct way to model Fe uptake rates by phytoplankton in ocean water, based on the following equation:

3 $${\mathrm{In}}\;{\mathrm{situ}}\;{\mathrm{uptake}}\;{\mathrm{rate}}\;({\mathrm{mol}}\; {\upmu}{\mathrm{m}}^{ - 2}{\mathrm{d}}^{ - 1}) =	\, {\mathrm{k}}_{{\mathrm{in - app}}}{\mathrm{/S}}{\mathrm{.A}}{\mathrm{.}}\;({\mathrm{L}}\;{\mathrm{\mu m}}^{ - 2}{\mathrm{d}}^{ - 1}) \\ 	\times \left[ {{\mathrm{SW}}\;{\mathrm{dissolved}}\;{\mathrm{Fe}}} \right]\left( {{\mathrm{mol}}\;{\mathrm{L}}^{ - 1}} \right)$$

where k_in-app_/S.A. can be modeled as a single averaged value (3.6 ± 0.25 × 10^−11^ L µm^−2^ d^−1^) or can be modified according to photochemistry and temperature. Equation 3 can be extended to include cell numbers or carbon so that in situ uptake rates appear in a more standardized form as uptake rate per cell or algal carbon, respectively. Note that the k_in-app_/S.A. obtained in this study is relevant only under Fe limitation, when phytoplankton maximize their Fe transport systems, and cell densities are low. Our study did not include Fe-limited cyanobacteria, which were reported to share similar k_in_/S.A. of Fe′ with eukaryotic phytoplankton, but dissimilar k_in_/S.A. of FeDFB or other Fe-siderophore complexes compared to eukaryotic phytoplankton [16, 50]. Naturally, more research is needed to extend our findings to cyanobacteria, and to fully understand the effect of speciation, photochemistry, and temperature on oceanic dFe bioavailability.

Currently the parameterization of dFe bioavailability in the biogeochemical model is lacking [51]. Better representation of iron control on phytoplankton growth in models is crucial and timely as it bares implications for the oceanic uptake of atmospheric CO_2_, nutrient cycles, ocean food webs, and eventually fish catch. The methodology presented here is simple and could assist in evaluating Fe bioavailability in many oceanic locations and under future conditions, including ocean acidification, warming, and deoxygenation. The straightforward approach for modeling in situ Fe uptake by phytoplankton based on dFe concentrations (Eq. 3) is particularly valuable, given the high-quality and high-resolution dFe data that are emerging on a global scale from the international GEOTRACES program [52].

## Supplementary information

Supplemental Material
