# Supplementary material for: Insights into the bioavailability of oceanic dissolved Fe from phytoplankton uptake kinetics
Source: ISME J. 2020 Feb 5;14(5):1182–93. doi: 10.1038/s41396-020-0597-3 (PMC7174416; doi:10.1038/s41396-020-0597-3)
Supplement: Supplementary file 1 — Supplemental Material [file 41396_2020_597_MOESM1_ESM.pdf]

Supplementary Information for the manuscript

Insights into the bioavailability of oceanic dissolved Fe from phytoplankton uptake kinetics

Yeala Shaked, Kristen N. Buck, Travis Mellett, and Maria. T. Maldonado

Content

Tables

Table S1. Values and errors of measured and calculated variables for the different water types

Table S2. Uptake experiments for determining Baffin Bay water dFe availability

Table S3. Comparison between calculated FeL dissociation rates and measured uptake rates

Table S4. Summary of uptake experiments for determining dFe availability in some of the water samples

Table S5. Surface area normalized uptake rate constants - k_{in-app}/S.A. (L μm^{-2} d^{-1}) of dFe in all samples as measured by the different Fe-limited phytoplankton species

Table S6. Compiled results and conditions of outdoor iron uptake experiments conducted to test the influence of photochemistry on dFe availability (k_{in-app}/S.A.).

Table S7. Calculation of the complexation capacity of free cell transport ligands

Figures

Figure S1. Relationships between Fe concentration and speciation parameters and dFe bioavailability indifferent seawater types

Figure S2. Equilibration between ^{55}FeEDTA and natural ligands as tested with CSV

References

Table S1. Values and errors of measured and calculated variables for the different water types

Samples	[dFe]* (nM)	$[L_1]$ (nM)	log K_1	$[L_2]$ (nM)	log K_2	$k_{in\text{-}app}$/S.A. L µm^{-2} d^{-1}	α'	[Fe'] pM	L_{tot} (nM)	eL (nM)	α
Baffin Bay- BBA	1.56	1.83 *(0.07)*	12.46 *(0.10)*	----	----	2.9E-11 *(2.7E-12)*	779	2.0	1.8	0.3	5278
Canadian Arctic Archipelago- CCA	0.97	1.79 *(0.07)*	12.06 *(0.07)*	----	----	1.4E-11 *(2.0E-12)*	941	1.0	1.8	0.8	2055
Beaufort Sea - BS	0.74	----	----	1.74 *(0.16)*	11.67 *(0.11)*	1.7E-11 *(4.1E-12)*	468	1.6	1.7	1.0	814
North Pacific - P16	0.58	----	----	1.83 *(0.16)*	11.69 *(0.09)*	4.9E-11 *(5.8E-12)*	612	0.9	1.8	1.3	896
North Pacific - P20_Surface	0.95	1.35 (0.09)	12.3 *(0.15)*	1.82 *(0.20)*	11.28 *(0.10)*	2.7E-11 *(4.5E-12)*	1145	0.8	3.2	2.2	3040
North Pacific - P20_Deep	0.97	----	----	3.23 *(0.44)*	10.91 *(0.13)*	3.9E-11 *(3.1E-12)*	184	5.3	3.2	2.3	263
Southern Ocean - SO	1.37	1.92	12.08	----	----	3.8E-11 *(6.9E-12)*	743	1.8	1.9	0.6	2308
Gulf of Mexico - GOM	0.60	----	----	1.81 *(0.24)*	11.79 *(0.18)*	3.8E-11 *(1.0E-11)*	746	0.8	1.8	1.2	1116
Atlantic Zonal Transect - GTNA	0.63	0.79 *(0.025)*	12.39 *(0.12)*	0.93 *(0.13)*	11.26 *(0.04)*	5.0E-11 *(9.2E-12)*	591	1.1	1.7	1.1	2108
Equatorial Pacific- EPZT_ODZ	1.67	1.48	12.57	2.73	11.08	2.9E-11 *(7.5E-12)*	302	5.5	4.2	2.5	5827
Equatorial Pacific- EPZT_Surface	0.46	0.78	12.35	1.22	11.39	5.4E-11 *(1.1E-11)*	955	0.5	2.0	2.1	2046
Equatorial Pacific- EPZT_Deep	1.17	----	----	3.01	11.39	5.7E-11 *(1.3E-11)*	513	2.3	3.0	1.8	739

1SD error for the different variables apear italized in paranthesis

* Ambeint dFe plus 0.4 nM ^{55}Fe

$\alpha' = \sum[eL_i] \times K^{cond}_{FeL_i,Fe'}$

$\alpha = \sum[L_i] \times K^{cond}_{FeL_i,Fe'}$

Table S2. Uptake experiments for determining Baffin Bay water dFe availability

Culture	Uptake rate mol cell^{-1} hr^{-1}	$K_{in\text{-}app}$ L cell^{-1} d^{-1}	Cell S.A. µm^{2} cell^{-1}	$K_{in\text{-}app}$/ S.A. L µm^{-2} d^{-2}	Cell density cell mL^{-1}	Total S.A.$^{\$}$ µm^{2} L^{-1}	Uptake rate pmol L^{-1} hr^{-1}	Total uptake* pmol L^{-1}
PM 1	7.5E-19	4.6E-08	1322	3.5E-11	4.9E+03	6.4E+06	8.8	61
PM 2	4.2E-19	2.9E-08	1322	2.2E-11	5.2E+03	6.9E+06	15	85
PP 1	3.5E-20	2.0E-09	49	4.1E-11	2.4E+05	1.2E+07	32	270
PP 2	1.8E-20	1.1E-09	50	2.3E-11	2.7E+05	1.4E+07	20	160
TO	3.6E-20	2.1E-09	84	2.5E-11	1.7E+05	1.4E+07	19	171
CP 1	2.9E-20	1.8E-09	51	3.5E-11	1.9E+05	9.9E+06	23	176
CP 2	2.0E-20	1.2E-09	54	2.3E-11	1.2E+05	6.4E+06	9.4	66
CP 3	1.3E-20	7.9E-10	53	1.5E-11	1.3E+05	7.1E+06	6.8	48
Mic	1.3E-21	1.1E-10	6	1.9E-11	1.6E+06	9.6E+06	11	73
Mic	1.2E-21	9.5E-11	6	1.6E-11	1.6E+06	9.5E+06	8.1	69
TW	2.4E-19	1.2E-08	392	3.1E-11	2.65E+04	1.0E+07	20	103

[dFe] = 1.56 nM (1.16 nM + 0.4nM ^{55}Fe)

$- Total surface area of cells = cell density x cell S.A.

* Overall Internalized Fe by the culture, based on the final time point in the uptake assay

Table S3. Comparison between calculated FeL dissociation rates and measured uptake rates

Samples	[dFe]*	log K_1	log K_2	k'_d (= k'_f/KFe'L)#		FeL dissociation rate@		Uptake rate	Estimated Fe' contribution to uptake*	
	(nM)	L_1	L_2	L_1 (s^{-1})	L_2 (s^{-1})	L_1 (pM hr^{-1})	L_2	pM hr^{-1}	Min (%)	Max (%)
Baffin Bay- BBA	1.56	12.46	----	2.4E-07		1.3		7 - 32	4	19
Canadian Arctic Archepelago-CCA	0.97	12.06	----	6.0E-07		2.1		5 - 13	16	42
Beaufort Sea - BS	0.74	----	11.67		6.9E-07		1.8	2 - 19		
North Pacific - P16	0.58	----	11.69		6.6E-07		1.4	2 - 21	7	69
North Pacific - P20_Surface	0.95	12.30	11.28					5 - 12		
North Pacific - P20_Deep	0.97	----	10.91		4.0E-06		14	5 - 17	82	278
Southern Ocean- SO	1.37	12.08	----	5.8E-07		2.8		5 - 28	10	57
Gulf of Mexico - GOM	0.60	----	11.79		5.2E-07		1.1	4 - 9	13	28
Atlantic Zonal Transect -GTNA	0.63	12.39	11.26					6 - 13		
Equatorial Pacific-EPZT_ODZ	1.67	12.57	11.08					9 - 21		
Equatorial Pacific-EPZT_Surface	0.46	12.35	11.39					6 - 10		
Equatorial Pacific-EPZT_Deep	1.17	----	11.39		1.3E-06		5.5	14 - 25	22	40

# - Constants from Croot and Heller 2012 (Table 1) for k'_d calculation:

	Low range (strong L ~L_1) k'_f	6.92E+05	(M^{-1} s^{-1})
	Low range (weak L ~L_2) k'_f	3.23E+05	(M^{-1} s^{-1})

@ - Dissociation rate of FeL (which equals formation rate of Fe') = k'_d x [dFe]

Table S4. Summary of uptake experiments for determining dFe availability in some of the water samples

Uptake experiments for determining Line P, Station P16, surface water (25m) dFe availability

Culture	Uptake rate	K_{in-app}	Cell S.A.	K_{in-app}/ S.A.	Cell density	Total S.A.$	Uptake rate	Total uptake*
	mol $cell^{-1}$ hr^{-1}	L $cell^{-1}$ d^{-1}	μm^2 $cell^{-1}$	L μm^{-2} d^{-2}	cell mL^{-1}	μm^2 L^{-1}	pmol L^{-1} hr^{-1}	pmol L^{-1}
PM	4.1E-19	2.7E-08	1000	2.7E-11	2.0E+03	2.0E+09	1.5	6
CP	4.6E-20	3.0E-09	51	5.9E-11	1.6E+05	8.0E+09	11	35
TP	6.7E-20	4.4E-09	70	6.3E-11	2.1E+05	1.4E+10	21	72
TW	3.1E-19	1.5E-08	392	3.7E-11	2.9E+04	8.1E+09	12	57
TW	4.3E-19	2.3E-08	392	5.8E-11	2.1E+04	1.1E+10	13	52
TW	1.5E-19	1.8E-08	364	4.9E-11	9.7E+03	3.5E+09	2.8	14

[dFe] = 0.58 nM (0.28 nM + 0.4 nM ^{55}Fe)

$- Total surface area of cells = cell density x cell S.A.

* Overall Internalized Fe by the culture, based on the final time point in the uptake assay

Uptake experiments for determining Line P, Station P20, deep water (800m) dFe availability

Culture	Uptake rate	K_{in-app}	Cell S.A.	K_{in-app}/ S.A.	Cell density	Total S.A.$	Uptake rate	Total uptake*
	mol $cell^{-1}$ hr^{-1}	L $cell^{-1}$ d^{-1}	μm^2 $cell^{-1}$	L μm^{-2} d^{-2}	cell mL^{-1}	μm^2 L^{-1}	pmol L^{-1} hr^{-1}	pmol L^{-1}
PP	4.1E-20	2.3E-09	58	4.0E-11	1.4E+05	8.0E+09	13	40
Mic	1.2E-20	8.0E-10	16	4.9E-11	1.7E+05	2.7E+09	5.3	40
CP	2.7E-20	1.7E-09	51	3.4E-11	1.8E+05	6.1E+09	8.3	66
TP	4.0E-20	2.2E-09	70	3.1E-11	1.2E+05	1.3E+10	17	58
TW	3.8E-19	1.5E-08	369	4.2E-11	1.9E+04	6.8E+09	14	41

[dFe] = 0.97 nM (0.57 nM + 0.4 nM ^{55}Fe)

$- Total surface area of cells = cell density x cell S.A.

* Overall Internalized Fe by the culture, based on the final time point in the uptake assay

Uptake experiments determining Line P, Station P20, surface water (25m) dFe availability

Culture	Uptake rate	K_{in-app}	Cell S.A.	K_{in-app}/ S.A.	Cell density	Total S.A.$	Uptake rate	Total uptake*
	mol $cell^{-1}$ hr^{-1}	L $cell^{-1}$ d^{-1}	μm^2 $cell^{-1}$	L μm^{-2} d^{-2}	cell mL^{-1}	μm^2 L^{-1}	pmol L^{-1} hr^{-1}	pmol L^{-1}
Mic	7.0E-21	7.1E-10	16	4.36E-11	1.9E+05	3.2E+09	4.5	9
CP	1.4E-20	8.8E-10	51	1.72E-11	1.5E+05	7.8E+09	5.2	17
TO	2.5E-20	1.8E-09	74	2.40E-11	1.7E+05	1.3E+10	11	34
TW	1.167E-19	1.1E-08	392	2.79E-11	4.1E+04	1.6E+10	12	57
TP	1.8E-20	1.6E-09	70	2.31E-11	2.1E+05	1.3E+10	4.6	41

[dFe] = 0.95 nM (0.55 nM + 0.4 nM ^{55}Fe)

$- Total surface area of cells = cell density x cell S.A.

* Overall Internalized Fe by the culture, based on the final time point in the uptake assay

Table S5. Surface area normalized uptake rate constants - $k_{in\text{-}app}$/S.A. (L µm^{-2} d^{-1}) of dFe in all samples as measured by the different Fe-limited phytoplankton species

Samples / Species	BBA	CAA	BS	P16	P20_Surf	P20_Deep	SO	GOM	GTNA	EPZT_ODZ	EPZT_Surf	EPZT_Deep	Average	SD	SD (%)
**TO**	2.5E-11	9.0E-12<br>9.3E-12	1.6E-11<br>1.3E-11		2.4E-11		5.0E-11	2.6E-11<br>6.6E-11	3.6E-11	2.7E-11	2.6E-11	4.5E-11	**2.9E-11**	1.7E-11	59
**PP**	4.1E-11<br>2.3E-11	1.9E-11	1.1E-11<br>1.2E-11			4.0E-11		1.3E-11	5.0E-11		6.3E-11	3.0E-11	**3.0E-11**	1.8E-11	59
**CP**	2.3E-11<br>3.5E-11<br>1.5E-11	1.7E-11		5.9E-11	1.7E-11	3.4E-11	1.6E-11			1.8E-11			**2.7E-11**	1.5E-11	54
**Mic**	1.6E-11<br>1.9E-11				4.4E-11	4.9E-11	4.6E-11	6.8E-11	7.9E-11	2.0E-11	7.9E-11	9.3E-11	**5.1E-11**	2.8E-11	54
**PM**	3.5E-11<br>2.2E-11			2.7E-11			2.8E-11	4.2E-11	5.0E-11	5.0E-11	7.1E-11	8.4E-11	**4.5E-11**	2.1E-11	46
**TW**	3.1E-11	1.4E-11	3.3E-11	5.8E-11<br>3.7E-11<br>4.9E-11	2.8E-11	4.2E-11			6.9E-11<br>1.6E-11			3.5E-11	**3.6E-11**	1.7E-11	47
**TP**				6.3E-11	2.3E-11	3.1E-11	5.0E-11	1.2E-11			3.1E-11		**3.5E-11**	1.9E-11	53

Table S6: Compiled results and conditions of outdoor iron uptake experiments conducted to test the influence of photochemistry on dFe availability ($k_{in\text{-}app}$/S.A.).

Illumination	Water type	Species or Cell Size	$k_{in\text{-}App}$/S.A. (L µm^{-2} d^{-1})	Sunlight/Dark
**Current study**	(Conditions- 2-3 hrs, 24-26°C, 0.4 nM ^{55}Fe)			
Sunlight<br>Dark	P20_Surf	*Thalassiosira oceanica*	5.1E-10<br>1.6E-10	3.1
Sunlight<br>Dark	P20_Deep	*Thalassiosira pseudonana*	5.0E-10<br>4.7E-10	1.1
Sunlight<br>Dark	GOM	*Thalassiosira weissflogii*	8.8E-10<br>6.6E-11	13
Sunlight<br>Dark	P16 (Exp 1)	*Thalassiosira oceanica*	6.8E-10<br>2.0E-10	3.4
Sunlight<br>Dark	P16 (Exp 2)	*Thalassiosira weissflogii*	6.7E-10<br>4.9E-11	13
Sunlight	GTNA	*Thalassiosira oceanica*	4.8E-10	2.5*
Sunlight	CCA	*Thalassiosira oceanica*	7.6E-10	4.0*
**Shi et al. 2010**	(Conditions- 1-2 hrs, 20°C, 0.5-1 nM ^{59}Fe)			
Artificial light	New Jersey Coast	*Thalassiosira weissflogii*	2.1E-10	NA
Sunlight	Atlantic (BATS)	*Thalassiosira weissflogii*	1.1E-09	NA
**Hassler & Schoemann 2009**	(Conditions - 16 hrs, 2°C, 1 nM ^{59}Fe)			
Artificial light	Southern Ocean	*Phaeocystis sp.*<br>*Chaetoceros sp.*	3.7E-10<br>2.3E-10	NA
**Maldonado et al. 2005**	(Conditions - 24-30 hrs, 14°C, 2nM ^{55}Fe)			
Sunlight<br>Dark	Southern Ocean	> 20 µm	5.8E-11<br>2.1E-12	27
Sunlight<br>Dark	Southern Ocean	2–20 µm	6.9E-11<br>5.6E-12	12
Sunlight<br>Dark	Southern Ocean	0.2–2 µm	6.5E-11<br>1.2E-11	5.2
**Mellett et al. 2018**	(Conditions - 7-9 hrs, 13°C, 0.2 nM ^{55}Fe)			
Sunlight<br>Dark	California Upwelling Zone	> 5 µm	2.8E-10<br>5.4E-11	5.2
Sunlight<br>Dark	California Upwelling Zone	1–5 µm	4.0E-10<br>6.8E-11	5.9
Sunlight<br>Dark	California Upwelling Zone	0.2–1 µm	6.5E-10<br>1.9E-10	3.4

Comments for Table S6

In most experiments some seawater samples were illuminated (noted as Sunlight or as Artificial light) while others were covered (noted as Dark).

Sunlight/Dark is the ratio between the uptake rate constants obtained in these conditions and represent the effect of photochemistry on dFe availability.

For CCA and GNTA which had only sunlight treatment, the ratios calculated using an averaged dark k_{in_app}/S.A. of 1.9E-10 L μm^{-2} d^{-1} (these are labelled with *)

Uptake rates per Carbon from Maldonado et al. 2005 were converted using cell radius of 15, 5 and 0.5 µm and cell Carbon content of 1E-10, 7E-12, and 2E-14 mol C $cell^{-1}$, respectively.

Uptake rates per *Chlorophyll a* (*Chl a*) from Mellett et al. 2018 were converted using cell radius of 5, 1.5 and 0.3 µm, cell Carbon content of 1E-12, 2E-13, 2E-15 mol C $cell^{-1}$ and Carbon to *Chl a* ratio of 50, 50 and 10 gC g*Chl a*$^{-1}$, respectively.

Table S7. Calculation of the complexation capacity of free cell transport ligands α_Y'

$\alpha_Y' = [Y] \times K'^{cond}_{FeY}$							
**Y** -	Cell-surface ligands-like components of Fe acquisition mechanisms						
**K'^{cond}_{FeY}** -	Conditional stability constant for Fe' binding by Y						
**α_Y'** in our		Minimal	**0.1**				
experiments		Maximal	**1.0**				
**Calculating [Y]**							
[Y] = cell surface Y desnity x total S.A in the experiment							
Cell-surface Y density of *Thalassiosira weissflogii (T.W)**						4E-20	molFe μm^{-2}
	(from Hudson and Morel, 1990)						
Total cell surface area in our experiments					Minimal	2E+09	$\mu m^2\ L^{-1}$
					Maximal	3E+10	$\mu m^2\ L^{-1}$
**[Y]** in our		Minimal	6E-11 mol Fe L^{-1}				
experiments		Maximal	1E-09 mol Fe L^{-1}				
*- Morel and Hudson 1990 report close values for additional phytoplankton							
**Calculating K'^{cond}_{FeY}**							
**$K'^{cond}_{FeY} = k_f / k^{FeY}_d$**							
**k_f -**	forward cosntant for Fe' binding to Y, measured for T.W*					9E+05	mol $L^{-1}\ s^{-1}$
	(from Hudson and Morel, 1990)						
**k^{FeY}_d** -	disccoiation rate constant of FeY, measured for T.W*					1E-03	s^{-1}
	(from Hudson and Morel, 1990)						
**K'^{cond}_{FeY}**	for T.W		9E+08 mol L^{-1}				

For this calculation we assume pseudo-equilibrium of Fe uptake and speciation.
This is a reasoanable assumption given our low cell densities, low light conditions and the presence of excess, strong organic Fe ligands

* Morel and Hudson 1990 report close values for additional phytoplankton

Figure S1: Relationships between dFe bioavailability of different seawater types and speciation parameters.

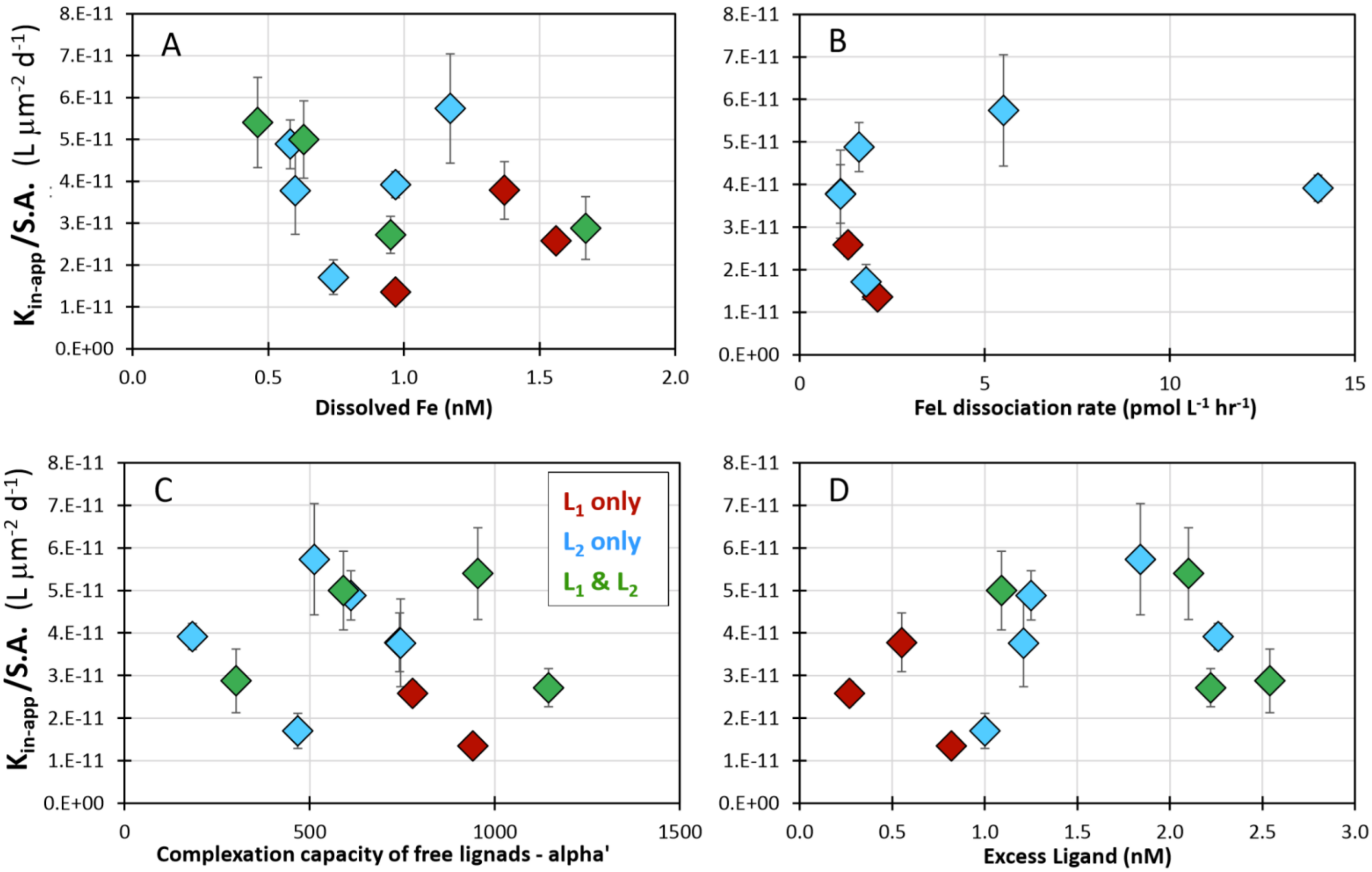

Figure S2: Equilibration between ^{55}FeEDTA and natural ligands

The equilibration between the added ^{55}Fe (added as ^{55}FeEDTA) and natural ligands is a critical aspect in our methodology. A complete equilibration is required so that phytoplankton can indeed access ^{55}FeL and ^{55}Fe’ in the uptake experiments and not ^{55}FeEDTA. We allowed 24 hrs for equilibration in the experiments. In a former study, however, a period of 2 hrs was shown as sufficient for equilibration between FeEDTA and natural ligands in the seawater (Mellett et al. 2018). These authors added 0.2 nM Fe bound to 0.3 nM EDTA to a seawater sample from the NE Pacific, and characterized Fe speciation before and after the addition by voltammetry. They observed a drop in measured excess ligand concentrations after the addition and an increase in the peak heights in the titration, consistent with a small Fe addition. Thus, these speciation measurements demonstrate an exchange of the added Fe from the FeEDTA with the natural ligand pool in the sample.

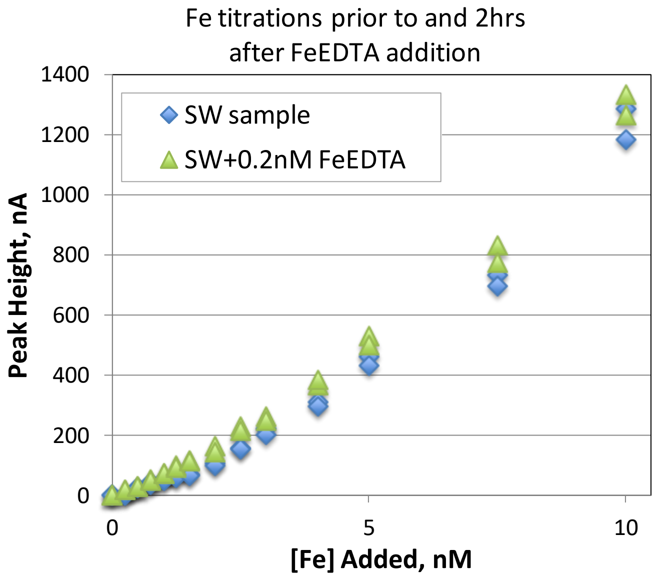

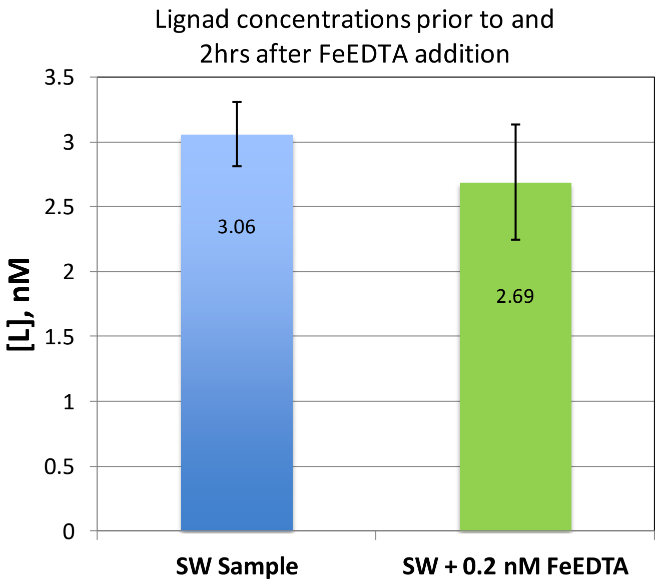

References:

1. Strzepek RF, Maldonado MT, Higgins JL, Hall J, Safi K, Wilhelm SW, et al. Spinning the "Ferrous Wheel": The importance of the microbial community in an iron budget during the FeCycle experiment. Global Biogeochem Cycles. 2005;19(4).

2. Mellett T, Brown MT, Chappell PD, Duckham C, Fitzsimmons JN, Till CP, et al. The biogeochemical cycling of iron, copper, nickel, cadmium, manganese, cobalt, lead, and scandium in a California Current experimental study. Limnol Oceanogr. 2018;63:S425–47.

3. Shi D, Xu Y, Hopkinson BM, Morel FMM. Effect of ocean acidification on iron availability to marine phytoplankton. Science. 2010;327(5966):676–9.

4. Hudson RJ., Morel FMM. lron transport in marine phytoplankton: Kinetics of cellular and medium coordination reactions. Limnol Oceanogr. 1990;35(5):1002–20.

5. Hassler CS, Schoemann V. Bioavailability of organically bound Fe to model phytoplankton of the Southern Ocean. Biogeosciences. 2009;6(10):2281–96.

6. Croot PL, Heller MI. The Importance of Kinetics and Redox in the Biogeochemical Cycling of Iron in the Surface Ocean. Front Microbiol. 2012;3(219).
